# Antioxidants Halt Axonal Degeneration in a Mouse Model of X-Adrenoleukodystrophy

**DOI:** 10.1002/ana.22363

**Published:** 2011-07

**Authors:** Jone López-Erauskin, Stéphane Fourcade, Jorge Galino, Montserrat Ruiz, Agatha Schlüter, Alba Naudi, Mariona Jove, Manuel Portero-Otin, Reinald Pamplona, Isidre Ferrer, Aurora Pujol

**Affiliations:** 1 Neurometabolic Diseases Laboratory, The Bellvitge Institute of Biomedical Research (IDIBELL), Hospitalet de LiobregatBarcelona, Spain; 2 The Centre Biomedical Network Research on Rare Diseases (CIBERER), The Spanish Institute for Health Carlos III (ISCIII)Barcelona, Spain; 3 Experimental Medicine Department, University of Lieida-IRB-LLEIDA (Biomedical Research Institute of Lieida)Lieida, Spain; 4 Neuropathology Institute, University Hospital of Bellvitge, University of BarcelonaBarcelona, Spain; 5 The Centre for Biomedical Network Research on Neurodegenerative Diseases (CIBERNED), The Spanish Institute for Health Carlos III (ISCIII)Barcelona, Spain; 6 Catalan Institution of Research and Advanced StudiesBarcelona, Spain

## Abstract

**Objective:**

Axonal degeneration is a main contributor to disability in progressive neurodegenerative diseases in which oxidative stress is often identified as a pathogenic factor. We aim to demonstrate that antioxidants are able to improve axonal degeneration and locomotor deficits in a mouse model of X-adrenoleukodystrophy (X-ALD).

**Methods:**

X-ALD is a lethal disease caused by loss of function of the ABCD1 peroxisomal transporter of very long chain fatty acids (VLCFA). The mouse model for X-ALD exhibits a late onset neurological phenotype with locomotor disability and axonal degeneration in spinal cord resembling the most common phenotype of the disease, adrenomyeloneuropathy (X-AMN). Recently, we identified oxidative damage as an early event in life, and the excess of VLCFA as a generator of radical oxygen species (ROS) and oxidative damage to proteins in X-ALD.

**Results:**

Here, we prove the capability of the antioxidants N-acetyl-cysteine, α-lipoic acid, and α-tocopherol to scavenge VLCFA-dependent ROS generation in vitro. Furthermore, in a preclinical setting, the cocktail of the 3 compounds reversed: (1) oxidative stress and lesions to proteins, (2) immunohistological signs of axonal degeneration, and (3) locomotor impairment in bar cross and treadmill tests.

**Interpretation:**

We have established a direct link between oxidative stress and axonal damage in a mouse model of neurodegenerative disease. This conceptual proof of oxidative stress as a major disease-driving factor in X-AMN warrants translation into clinical trials for X-AMN, and invites assessment of antioxidant strategies in axonopathies in which oxidative damage might be a contributing factor. Ann Neurol 2011;

Oxidative stress has been said to participate in the onset and/or progression of neurodegeneration in human neurological diseases of diverse etiology, including Parkinson disease, amyotrophic lateral sclerosis, multiple sclerosis, Alzheimer disease, and Huntington disease, to cite just a few.^1^–^5^ A common theme in all these diseases is axonal degeneration, which is seen preceding neuronal cell body's death, and might be responsible for much of the chronic disability.^6^,^7^ Axons are highly vulnerable, as their unusual size and high metabolic demands render them susceptible to injury, ischemia, transport defects, and oxidative damage. An indirect link between oxidative stress and axonal damage in vitro or ex vivo^8^ has been suggested in chemically induced models of oxidative injury.^9^,^10^ However, a causative role for oxidative stress in axonal degeneration in mouse models relevant to human disease has, to the best of our knowledge, not yet been formally proven.

To address this question, we chose a mouse knockout lacking ABCD1, a peroxisomal transporter of very long-chain fatty acids (VLCFA). This is the murine model of X-linked adrenoleukodystrophy (X-ALD: McKusick No. 300100), a rare and fatal disease characterized by central inflammatory demyelination within the central nervous system or slowly progressive spastic paraparesis, as a consequence of axonopathy in the spinal cord.^11^–^13^ X-ALD is the most frequently inherited leukodystrophy, with a minimum incidence of 1 in 17,000 males. The gene mutated in the disease encodes the ABCD1 protein, an adenosine triphosphate-binding cassette peroxisomal transporter involved in the import of very long chain fatty acids (C · 22:0) and VLCFA-CoA esters into the peroxisome for degradation.^14^,^15^ Defective function of the ABCD1 transporter leads to VLCFA accumulation in most organs and plasma; elevated levels of VLCFA are used as a biomarker for the biochemical diagnosis of the disease. Classical inactivation of ABCD1 in the mouse results in late onset neurodegeneration with axonopathy in the spinal cord that, in the absence of inflammatory demyelination in the brain, resembles the most frequent X-ALD phenotype or adrenomyeloneuropathy.^16^,^17^ Oxidative damage has been evidenced in postmortem brain samples from individuals with cerebral ALD^18^,^19^ and in mouse spinal cord prior to disease onset.^20^ The source of this oxidative damage is possibly related to the excess of saturated and unsaturated VLCFA, shown to generate both free radicals and oxidative damage to proteins in vitro.^20^,^21^

In the present study, we set out to test the potential of 3 well-known antioxidants, α-tocopherol, N-acetylcysteine (NAC), and α-lipoic acid (LA), first to scavenge VLCFA-dependent reactive oxygen species production, and then to ameliorate the neurodegenerative adrenomyeloneuropathy (AMN)-like phenotype observed in mouse models of X-ALD. The 3 substances are US Food and Drug Administration (FDA)-approved drugs shown to be able to cross the brain–blood barrier and to achieve neuroprotective effects in mouse models of neurodegeneration, although their specific effect on axonal degeneration has not been addressed.^22^–^24^

## Materials and Methods

### Chemicals

The following chemicals were used: 6-carboxy-2′, 7′-dichlorodihydrofluorescein diacetate, diacetoxymethyl-ester (H_2_-DCFDA) (Invitrogen, Carlsbad, CA), hexacosanoic acid, C26:0 (Sigma, St Louis, MO), NAC (Sigma), LA (Sigma), and Trolox (Calbiochem, San Diego, CA).

### Mouse Breeding

The generation and genotyping of *Abcd1*^−^ mice have been previously described.^16^,^17^,^25^ Mice used for experiments were of a pure C57BL/6J background, all male. Animals were sacrificed, and tissues were recovered and conserved at −80°C. All methods employed in this study are in accordance with the Guide for the Care and Use of Laboratory Animals published by the US National Institutes of Health (NIH Publication No. 85-23, revised 1996), and with the ethical committee of The Bellvitge Institute of Biomedical Research and the government of Catalonia.

### Antioxidant Dosage and Treatment of Mice

LA (0.5% wt/wt) and α-tocopherol (1050IU/kg in food) were mixed into AIN-76A chow from Dyets (Bethlehem, PA).^24^,^26^ NAC (1%) was dissolved in water (pH 3.5).^22^ Doses for NAC, LA, and vitamin E were, respectively, 850mg/kg/day, 430 mg/kg/day, and 90IU/kg/day (65mg/kg/day). Equivalent doses for patients have been calculated using the FDA-recommended scaling factors for a first use in patients (Guidance for Industry: Estimating the Maximum Safe Starting Dose in Initial Clinical Trials for Therapeutics in Adult Healthy Volunteers; http://www.fda.gov/downloads/Drugs/GuidanceComplianceRegulatoryInformation/Guidances/ucm078932.pdf). For a 70kg patient, equivalent doses would be 4.8g/day for NAC, 2.4g/day for LA, and 510IU/day (369mg/day) for vitamin E. Similarly high doses have already been given to patients in chronic treatments, with minimal to no side effects reported.^27^–^30^ Combinations of the antioxidants have also been given without significant interactions or side effects described, although at lower doses.^31^,^32^

For locomotor tests and immunohistological analysis, 12-month-old animals were randomly assigned to 1 of the following dietary groups for 6 months. Group I (wild-type [Wt]) mice (n = 12) received only normal AIN-76A chow, Group II (Wt + antioxidants [Antx]) Wt mice (n = 9) were treated with chow containing LA and α-tocopherol and with NAC in drinking water, Group III *Abcd1*^*−*^*/Abcd2*^*−/−*^ (Dko) mice (n = 17) received only normal AIN-76A chow, and Group IV (Dko + Antx) *Abcd1*^*−*^*/Abcd2*^*−/−*^ mice (n = 12) were treated with chow containing LA and α-tocopherol, and with NAC in drinking water.

For evaluation of oxidative damage and neuropathology, 16-month-old animals were randomly assigned to 1 of the following dietary groups for 6 months. Group I mice (Wt) (n = 8) were fed normal AIN-76A chow, Group II mice (Wt + Antx) (n = 8) were treated with the antioxidants as above, Group III mice (*Abcd1*^*−*^) (n = 8) were fed normal AIN-76A chow, and Group IV mice (*Abcd1*^*−*^) (n = 8) were treated with antioxidant cocktail as above.

### Cell Culture and Treatments

Control and X-ALD human fibroblasts were grown as described.^20^ Intracellular ROS levels were estimated using the ROS-sensitive H_2_DCFDA probe as described.^20^ Detailed methodology is described in Supplementary Methods.

Control (n = 5) and X-ALD human fibroblasts (n = 5) were treated in medium containing fetal calf serum (10%) for 24 hours with ethyl alcohol as control, C26:0 (50μM), or antioxidant with C26:0 (50μM). Three different antioxidants were used at doses previously reported in fibroblasts: Trolox (2mM),^33^ NAC (1mM),^34^,^35^ and LA (0.5mM)^34^–^36^; for the higher doses, Trolox was used at 500nM, NAC at 50μM and LA at 50μM. The maximum concentration of ethanol used was 2.2%. Ethanol does not produce ROS by itself (data not shown). Experiments were carried out with cells at 95% of confluence, which had a number of passages ranging from 12 to 15.

### Evaluation of Oxidative Lesions

*N*^ɛ^-(carboxymethyl)-lysine (CML), *N*^ɛ^-(carboxyethyl)-lysine (CEL), and *N*^ɛ^-malondialdehyde-lysine (MDAL) concentrations in total proteins from spinal cord homogenates or human fibroblasts were measured with gas chromatography/mass spectrometry (GC/MS), as reported.^20^ The amounts of products were expressedas the ratio of micromole of glutamic semialdehyde, aminoadipicsemialdehyde, CML, CEL, or MDAL/mol of lysine. Evaluation of direct carbonylation has been performed as previously described.^37^ Detailed methodology is described in Supplementary Methods.

### Immunohistochemistry

Spinal cords were harvested from 22-month-old Wt, *Abcd1*^*−*^, and *Abcd1*^*−*^ mice fed with the cocktail of antioxidants for 6 months, after perfusion with 4% paraformaldehyde as described.^16^,^38^ Detailed methodology is described in the Supplementary Methods.

### Behavioral Testing

#### Treadmill Test

The treadmill apparatus consisted of a variable speed belt varying in terms of speed and slope. An electrified grid was located to the rear of the belt on which foot shocks (0.2mA) were administered whenever the mice fell off the belt. The treadmill apparatus (Panlab, Barcelona, Spain) consisted of a belt (50cm long and 20cm wide) varying in terms of speed (5–150cm/s) and slope (0–25°) enclosed in a Plexiglas chamber.^39^ The latency to falling off the belt (time of shocks in seconds) and the number of received shocks were measured. For detailed protocol, see Supplementary Methods.

#### Horizontal Bar Cross Test

Bar cross test was performed as previously described.^38^ Detailed methodology is described in Supplementary Methods.

### Statistical Analyses

Data are given as mean ± standard deviation. Significant differences were determined by 1-way analysis of variance followed by Tukey Honestly Significant Difference post-test after verifying normality. For Figure 4G, the number of mice able to perform the treadmill test was counted and represented as a percentage of mice. Significant differences were determined by chi-square test. Statistical analyses were performed using the SPSS 12.0 program (SPSS Inc., Chicago, IL).

## Results

### α-Tocopherol, NAC, or LA Successfully Prevents Hexacosanoic Acid-Dependent ROS Generation In Vitro

A consequence of ROS production is their interaction with biomolecules, in particular DNA, lipids, and proteins, which are then modified and functionally altered. Proteins can be directly damaged by ROS in a process called carbonylation, or indirectly damaged by reaction with active aldehyde products of lipid peroxidation (eg, malondialdehyde [MDA] or hydroxynonenal) or with products of glycoxidation, (eg, glyoxal or methylglyoxal), or as a result of alterations in membrane lipid microenvironment secondary to the peroxidative process. In X-ALD, an increase of markers of lipoxidation (MDA-lysine), combined with markers of glycoxidation and lipoxidation, CEL and CML, together with markers of direct carbonylation, can be detected in spinal cords and in peripheral mononuclear cells or fibroblasts.^20^,^21^ Also, excess of VLCFA decreases reduced glutathione, and X-ALD cells are more sensitive to glutathione depletion.^20^ Thus, an ideal antioxidant strategy would combine different compounds acting through complementary mechanisms for ROS scavenging.

**Figure 1:**
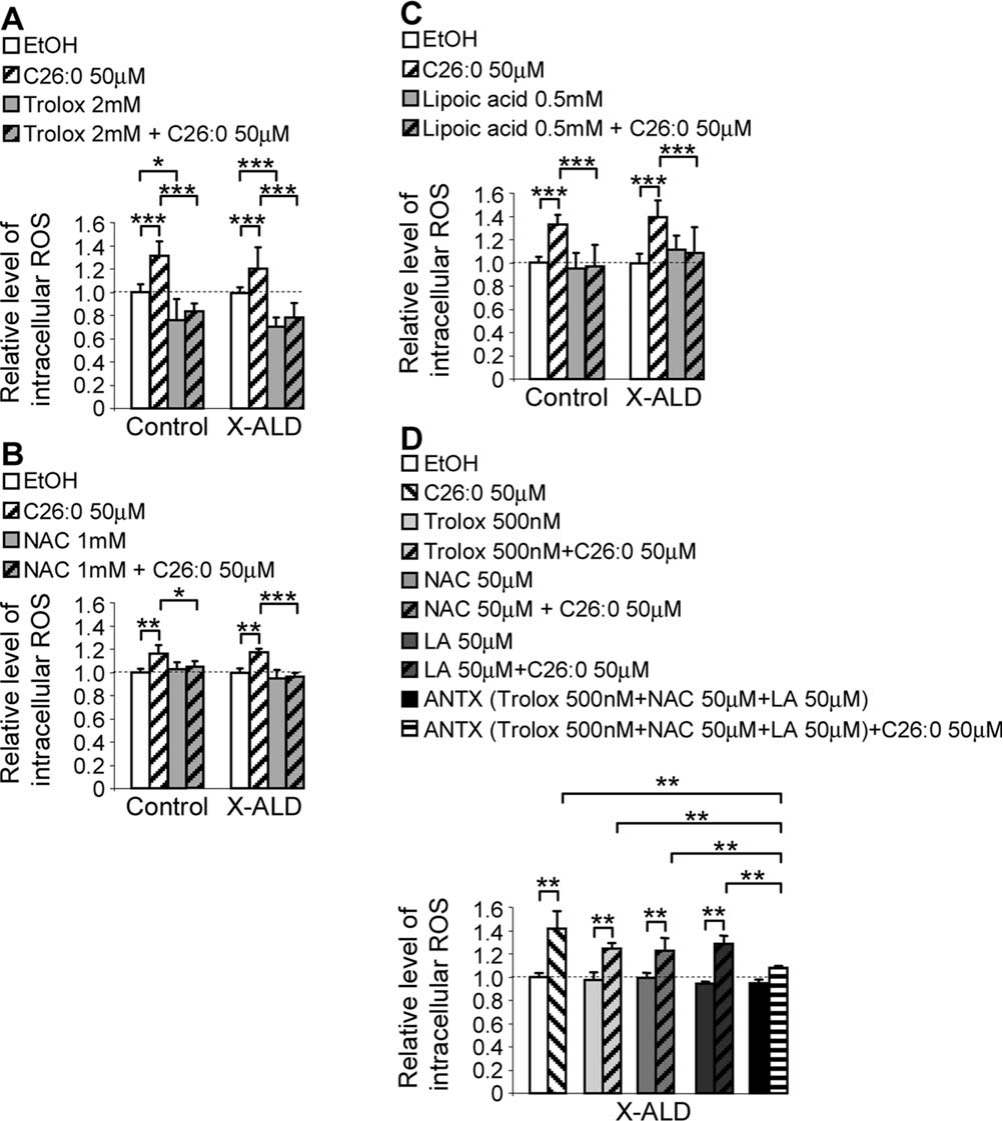
Trolox, N-acetylcysteine and α-lipoic acid (LA) prevent radical oxygen species (ROS) generated by C26:0. Intracellular ROS was measured in control (n = 5) and X-adrenoleukodystrophy (X-ALD) human fibroblasts (n = 5) after 24 hours. Three different antioxidants were used at high doses: (A) Trolox, (B) N-acetylcysteine (NAC), and (C) LA. (D) The 3 antioxidants were used alone or in combination at lower doses. Significant differences were determined as described in Materials and Methods (*p < 0.05, **p < 0.01, ***p < 0.001). EtOH = ethyl alcohol; ANTX = antioxidants.

We chose alpha tocopherol (in the form of its analogue Trolox),^40^ as it can inhibit the propagation phase of the peroxidative process by neutralizing the lipid-derived radicals; NAC, as it can regenerate reduced glutathione and scavenge several ROS, including OH, H_2_O_2_, peroxyl radicals, and nitrogen-centered free radical^41^; and LA, as it can regenerate glutathione from its oxidized counterpart (oxidized glutathione), ascorbate from dehydroascorbate, and α-tocopherol from tocopheryl radicals,^42^ thus enhancing the effects of the other 2 compounds. LA and its reduced form, dihydrolipoic acid, may use their chemical properties as a redox couple to alter protein conformations by forming mixed disulfides, thus protecting proteins from oxidation. We thus investigated the potential of the 3 agents to scavenge ROS production generated by an excess of hexacosanoic acid (C26:0), as measured using the probe dichlorofluorescein. All 3 antioxidants were capable individually of normalizing ROS levels after the addition of 50μM hexacosanoic acid at high but not at low doses (Fig 1). When combining the antioxidants at low doses, a synergistic effect was observed, resulting in a full prevention of ROS accumulation (see Fig 1D).

### Combination of α-tocopherol, NAC, and LA Blocks Oxidative Stress and Damage to Proteins and DNA in Spinal Cord From Abcd1^−^ Mice

**Figure 2:**
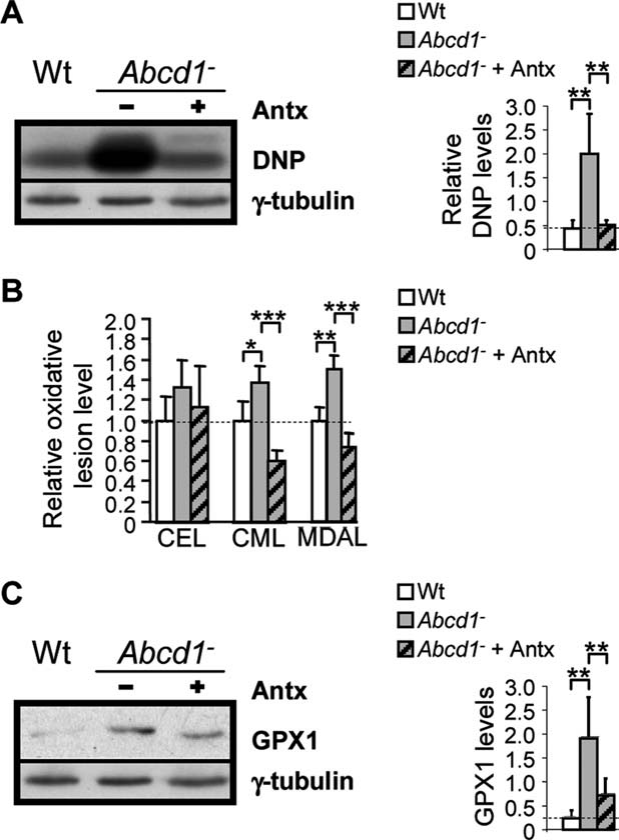
Antioxidant treatment normalizes oxidative lesions markers in spinal cord from 22-month-old *Abcd1*^*−*^ mice. (A) Dinitrophenol (DNP) levels in 22-month-old wild-type (Wt), *Abcd1*^*−*^, and *Abcd1*^*−*^ + antioxidants (Antx) mice. The quantification of these blots by densitometry was performed and normalized to γ-tubulin. (B) N^ɛ^-(carboxymethyl)-lysine (CML), N^ɛ^-(carboxyethyl)-lysine (CEL), and N^ɛ^-malondialdehyde-lysine (MDAL) in Wt, *Abcd1*^*−*^, and *Abcd1*^*−*^ + Antx mice. (C) GPX1 levels were quantified in Wt, *Abcd1*^*−*^, and *Abcd1*^*−*^ + Antx mice. Significant differences were determined as described in Materials and Methods (n = 6 mice per genotype and condition; *p < 0.05, **p < 0.01, ***p < 0.001).

**Figure 3:**
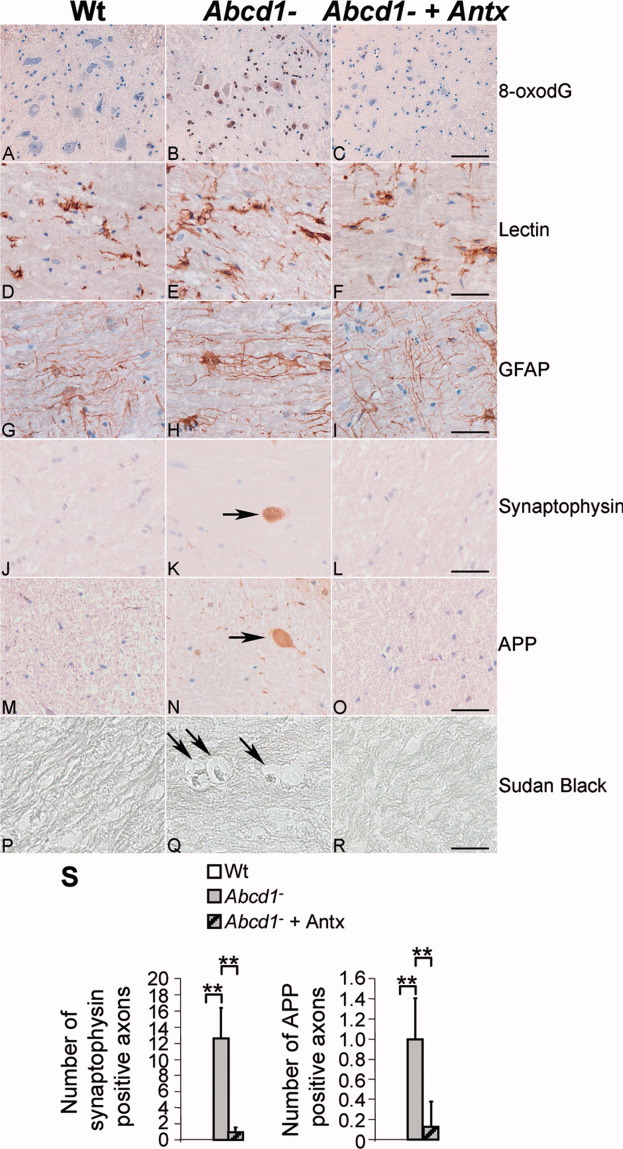
Oxidative stress, myelin, and axonal pathologies in 22-month-old *Abcd1*^*−*^ spinal cord are prevented by an antioxidant cocktail. Longitudinal sections of the dorsal spinal cord in wild-type (Wt) (A, D, G, J, M, P), *Abcd1*^*−*^ (B, E, H, K, N, Q) and *Abcd1*^*−*^ + antioxidants (Antx) (C, F, I, L, O, R) mice were processed for 8-oxo-7,8-dihydro-2′-deoxyguanosine marker (8-oxodG) (A–C), lectin *Lycopersicon* *esculentum* (D-F), glial fibrillary acidic protein (GFAP) (G–I), synaptophysin (J–L), amyloid precursor protein (APP) (M–O), and Sudan black (P–R). Bar = 25μm. (S) Quantification of APP and synaptophysin accumulation in axonal swellings in Wt, *Abcd1*^*−*^, and *Abcd1*^*−*^ + Antx mice. Significant differences were determined as described in Materials and Methods (n = 5–6 mice per genotype and condition; **p < 0.01).

Combined antioxidant therapy is aimed at reproducing the multistep, combined response that is observed in vivo leading to recovery after an oxidative challenge.^43^ Some studies have shown that combinations of antioxidants can be beneficial for pathologies associated with increased oxidative stress,^44^ and that such a strategy might be advantageous over higher doses of single antioxidants for treating mitochondriopathies,^45^,^46^ reproducing what is already present in nature, that is, a combination of antioxidant systems rather than a single system. Thus, we treated a group of *Abcd1*^−^ mice at disease onset (16 months old) with a mixture of the 3 antioxidants for 6 months, and compared spinal cord samples from Wt, *Abcd1*^*−*^, and *Abcd1*^*−*^ mice fed with antioxidants (*Abcd1*^*−*^ + Antx). We semiquantified carbonyl residues with an anti-dinitrophenol antibody^37^ to find a normalization of the amount of oxidized proteins when antioxidants were used (Fig 2A). Further, we quantified by GC/MS the levels of the markers of glycoxidative and lipoxidative lesions, which were also normalized owing to the antioxidant treatment (see Fig 2B). We had previously observed that the glutathione peroxidase enzyme GPX-1 is strikingly induced in *Abcd1*^*−*^ spinal cord, reflecting a physiological antioxidant response to increased oxidative stress.^20^ This increase was lowered upon treatment, suggesting that free radicals are scavenged by these compounds (see Fig 2C). As a consequence of oxidative stress, damage to DNA occurs and is of particular importance due to the possibility of producing mutations compromising cell survival or accelerating aging.^47^ We performed immunohistochemistry against the widely used 8-oxo-7,8-dihydro-2′-deoxyguanosine marker (8-oxodG)^48^ to observe an increase in labeling in several nuclei of motoneurons and interneurons (Fig 3A–C and Supplementary Table), thus pinpointing these neuronal subpopulations as plausible first targets for the damage to DNA produced by the oxidative stress. This finding is in agreement with the detected upregulation of catalase in these cell types, as described.^20^ The combination of antioxidants used successfully diminished the labeling of motoneurons and interneurons with 8-oxodG antibody as compared to untreated *Abcd1*^−^ mice (see Fig 3A–C and Supplementary Table).

### Treatment with α-Tocopherol, NAC, and LA Initiated after Disease Onset Rescues Axonal Degeneration in X-ALD Mouse Models

Furthermore, we investigated the effects of the treatment on the neurodegenerative phenotype exhibited by X-ALD mouse models. *Abcd1*^*−*^ mice present an overt neuropathological phenotype at 22 months of age, characterized by axonal damage, as suggested by the accumulation of amyloid precursor protein (APP) and synaptophysin in axonal swellings. This is accompanied by scattered myelin debris, as revealed by Sudan black, and astrocytosis and microgliosis, as identified with glial fibrillary acidic protein (GFAP) and lectin staining, respectively, without signs of apoptosis (see Fig 3D–S and Supplementary Table).^16^ The first abnormal deposition of synaptophysin is seen at around 12 months of age, earlier than astrogliosis. The most affected areas for both the axonal and the accompanying reactive glial changes are the pyramidal tracts and dorsal fascicles. After 6 months of antioxidant diet started at 16 months of age, we observed that axonal damage as measured by quantifying APP and synaptophysin deposition was strikingly reduced to control levels (see Fig 3D–S and Supplementary Table). Also, the number of reactive astrocytes and reactive microglia was reduced, but not the total numbers of astrocytes and microglia. These results suggest that oxidative damage control halts axonal degeneration in the mouse model used.

### Antioxidant Therapy Prevents and Arrests Progression of Locomotor Deficits in Abcd1^−^/Abcd2^−/−^ Mice

**Figure 4:**
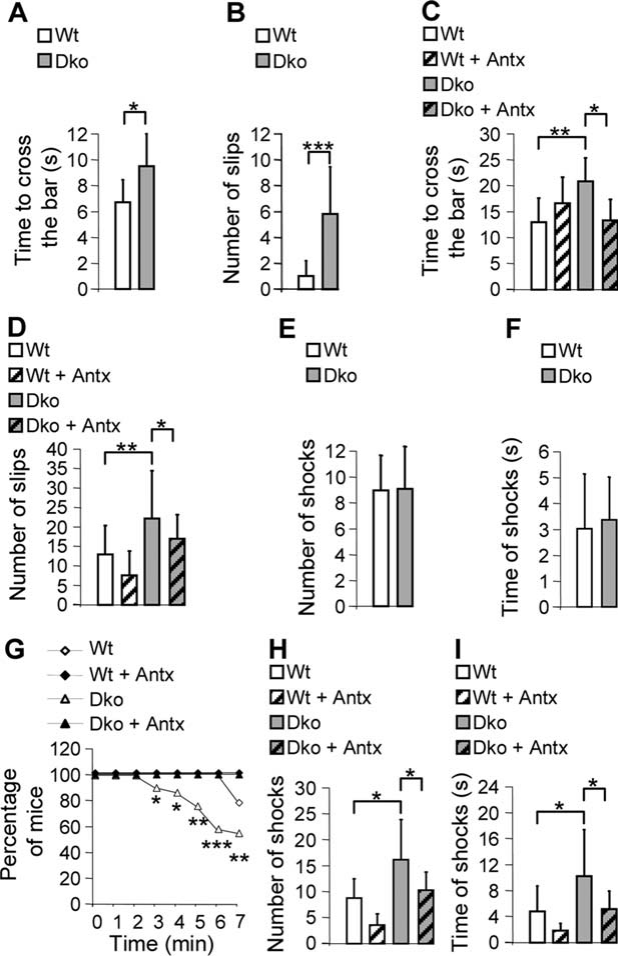
A combination of antioxidants rescues locomotor deficits in *Abcd1*^*−*^*/Abcd2*^*−/−*^ mice. Bar cross test (A–D) and treadmill experiment (E–I) were carried out at 12 and 18 months of age (wild-type [Wt; n = 12], Wt + antioxidants [Antx; n = 9], *Abcd1*^*−*^*/Abcd2*^*−/−*^ [Dko; n = 17], and *Abcd1*^*−*^*/Abcd2*^*−/−*^ + Antx [Dko + Antx; n = 12]). The time spent to cross the bar (A, C) and the number of slips (B, D) were quantified at 12 (A, B) and 18 months of age (C, D). Treadmill experiments were performed in Wt and Dko mice at 12 (E, F) and 18 months of age (G, I). Number of shocks (E, H) and latency to falling off the belt (time of shocks in seconds) (F, I) were quantified after 7 minutes. The percentage of mice still running/minute is represented panel G. Significant differences were determined as described in Materials and Methods (*p < 0.05, **p < 0.01, ***p < 0.001).

We chose a double mutant *Abcd1/abcd2*, a model in which the transporters of both homologs have been deleted by classical gene targeting.^16^,^17^,^38^,^49^ As the 2 proteins share overlapping functions in vivo in the metabolism of fatty acids,^38^,^50^ double mutants exhibit higher VLCFA accumulation in spinal cord,^16^ higher levels of oxidative damage to proteins,^21^ and a more severe AMN-like pathology, with an earlier onset than is the case with the single mutant *Abcd1*. Synaptophysin and abnormal accumulation of APP in damaged axons are the earliest immunohistological markers, evidenced from 12 months onward, at a level of pathology comparable to the 22-month-old *Abcd1* knockouts.^16^,^38^ Also, locomotor testing is facilitated in this model, as the first signs of neurological involvement can be seen at 15 months of age, using the bar cross test.^38^ For the sake of starting the treatment on symptomatic mice, we re-evaluated locomotor skills by using the bar cross^38^ and treadmill tests,^39^ starting at 12 months of age. Confirming previous results at 15 months, the *Abcd1/Abcd2* null mice presented abnormal scores as they required more time to reach a platform along the bar. Double mutants also exhibited a marked tendency to slip off the bar, as a sign of ataxia present in the pretreatment phase (Fig 4A, B). The treadmill test was not sensitive enough to detect abnormalities at that age, however (see Fig 4E, F).

We thus launched a preclinical trial with 4 groups of mice, Wt on vehicle or oral antioxidants, and double mutants on vehicle or antioxidants, and treated them for 6 months starting at 12 months of age. At the end of treatment, beneficial effects of antioxidants were striking, reaching full normalization of the time used to cross the bar and the number of slips (see Fig 4C, D and Supplementary Figs 1 and 2). Double mutants on normal chow presented with postural hypotonia and ataxia. Seventy to 80% of these double mutants had severe difficulties standing on their 4 limbs on the bar; they wrapped their hind and fore limbs around the bar instead, and used their fore limbs to drag themselves along the beam. Trembling was also very frequently noticed; these were features also visible in older (22–24 month) single *Abcd1* null mice, and might mirror the spastic paraparesis and ataxic gait that X-AMN patients suffer. These phenotypic abnormalities were absent in mice that received the antioxidant treatment. In the treadmill test, at a belt speed of 30cm/sec and 20° slope, differences were detected in *Abcd1/abcd2* compared to Wt mice. The number of mice that remained on the platform was recorded each minute. At minute 6, only 60% of *Abcd1/abcd2* mice were able to continue running, whereas all Wts were still on the treadmill (see Fig 4G). In addition, at the end of the experiment a higher cumulative number and duration of shocks (see Fig 4H, I) was observed in the mutant animals. Upon treatment, all *Abcd1/abcd2* mice were able to perform the test until the end, and were indistinguishable from Wts or from Wts fed with antioxidants regarding number of shocks and cumulative time (see Fig 4G–I). Immunohistological analysis demonstrated full recovery of axonal degeneration features as measured by APP and synaptophysin staining, and decrease of activated astrocytes and microglia (Supplementary Fig 3 and Supplementary Table). Taken together, our results provide compelling evidence for a beneficial effect of a long trial with a combination of α-tocopherol, NAC, and LA in reversing oxidative lesions at the cellular and tissue level, arresting axonal damage, and rescuing the locomotor neurological abnormalities in mouse models of X-ALD.

## Discussion

We have previously suggested that oxidative damage, because of its early appearance in the disease cascade and its direct relationship with the accumulation of VLCFA, could be a contributing factor to X-ALD disease pathogenesis.^20^ The work presented here indicates that the cocktail of antioxidants used efficaciously reverses the oxidative damage to proteins in whole spinal cords, and also specifically on DNA of spinal motoneurons and interneurons. Chronic DNA damage leads to accelerated aging,^47^ and is readily detected at 3.5 months of age in these cell types, together with increased catalase immunostaining,^20^ thus suggesting a plausible origin for the axonal degenerative process that is evidenced much later in life.

Furthermore, we present compelling evidences that the chosen antioxidant combination halted clinical progression and reversed axonal damage in the mouse model, thus providing the formal conceptual proof for oxidative injury or at least an antioxidant-sensitive process as a main etiopathogenic factor in this disease. This constitutes a novel finding, as oxidative stress and damage are involved in a wide variety of neurological diseases,^2^ and thus have been classically considered common and simply epiphenomenal events in the neurodegenerative cascade that occurs in the late stages of the disorders. This concept has been reinforced by the general lack of therapeutic effects of antioxidants in randomized clinical trials, which might indicate that oxidative stress would not constitute a major contributor to disease pathology, at least at the stage when the treatment was applied. However, exceptions can be made when the causality link between oxidative stress and disease is well established, as is the case for Friedreich ataxia, a rare disease caused by mutations in frataxin, a mitochondrial ferrosulfur protein involved in ROS homeostasis.^51^ Friedreich patients exhibit spinocerebellar ataxia with dorsal root ganglia degeneration.^52^,^53^ Clinical trials using high doses of the antioxidant idebenone, a coenzyme Q10-related molecule, have demonstrated that this compound is able to improve both cardiac hypertrophy and neurological symptoms associated with the disease.^54^,^55^ In contrast, low doses of idebenone failed to render neurological benefits in similar clinical settings,^56^ suggesting a critical issue regarding the appropriate dose, perhaps hampering positive outcomes in other studies.

Although externally added oxidant insults have been shown to produce axonal degeneration in culture,^10^ a disease-relevant murine model for axonal damage, caused by endogenously produced oxidative stress, was lacking until now. Thus, the *Abcd1* null mouse appears to be a useful model for dissecting the molecular and cellular changes underlying oxidative stress-dependent axonal pathology, in so doing providing insights into the cascade of events that cause irrevocable nerve cell degeneration. Importantly, the model provides a long window of opportunity for intervention, during which the initial axon dysfunction has not yet progressed to frank degeneration. Targeted strategies to ameliorate these axonal changes may provide a new approach to delaying the cascade of intracellular changes in other diseases with axonal damage.

Our data strongly suggest that an early and carefully tailored antioxidant intervention using the cocktail described could be a plausible therapeutic option for X-AMN patients, who do not suffer from severe neuroinflammatory demyelination. Biological effects could be easily monitored by quantitative measurement of biomarkers of oxidative damage in the peripheral blood mononuclear cells in X-AMN patients, as previously shown.^21^ Therapeutic implications derived from this work could be extrapolated to other diseases that share both axonal degeneration as a significant component of clinical progression and oxidative stress as a main or early contributing pathogenic factor.
