# Supplementary material for: Antioxidants Halt Axonal Degeneration in a Mouse Model of X-Adrenoleukodystrophy
Source: Ann Neurol. 2011 Jul;70(1):84–92. doi: 10.1002/ana.22363 (PMC3229843; doi:10.1002/ana.22363)
Supplement: Supplementary file 3 [file ana0070-0084-SD3.doc]

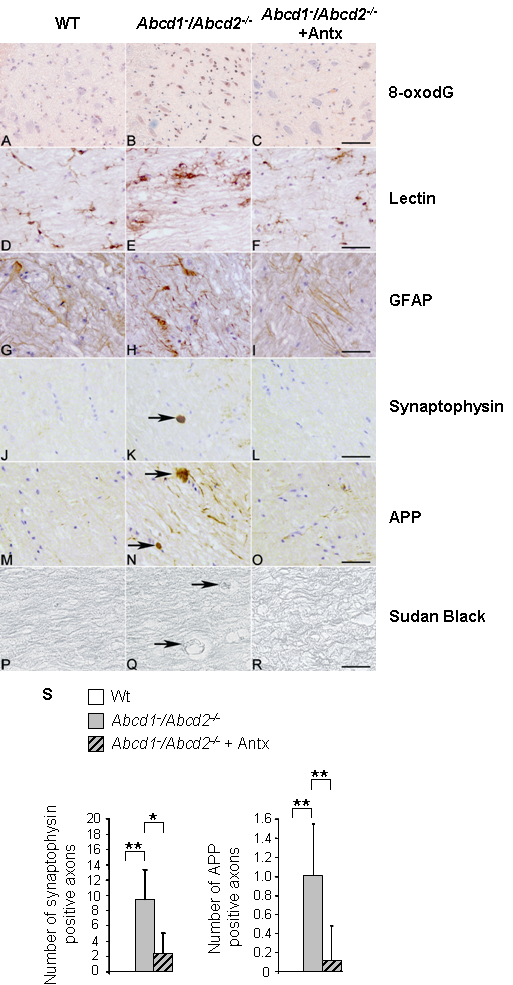


**Supplementary Figure 3. Oxidative stress, myelin and axonal pathologies in 18-month-old *Abcd1^-^/Abcd2^-/-^* spinal cord are rescued by antioxidant cocktail.** Longitudinal sections of the dorsal spinal cord in Wt (A, D, G, J, M, P), *Abcd1^-^/Abcd2^-/-^* (B, E, H, K, N, Q) and *Abcd1^-^/Abcd2^-/-^* + Antx (C, F, I, L, O, R) mice processed for 8-oxodG (A-C), lectin *Lycopericon esculentum* (D-F), GFAP (G-I), synaptophysin (J-L), APP (M-O) and Sudan black (P-R). Bar=25μm. S) Quantification of APP and synaptophysin accumulation in axonal swellings in Wt, *Abcd1^-^/Abcd2^-/-^* and *Abcd1^-^/Abcd2^-/-^* + Antx mice. Significant differences were determined as described in materials and methods (n=5-6 mice per genotype and condition; ^*^*P*<0.05, ***P*<0.01, ****P*<0.001).
