# Supplementary material for: Antioxidants Halt Axonal Degeneration in a Mouse Model of X-Adrenoleukodystrophy
Source: Ann Neurol. 2011 Jul;70(1):84–92. doi: 10.1002/ana.22363 (PMC3229843; doi:10.1002/ana.22363)
Supplement: Supplementary file 4 [file ana0070-0084-SD4.doc]

**SUPPLEMENTARY METHODS**

**Evaluation of intracellular ROS**

Intracellular ROS levels were estimated using the ROS-sensitive H_2_DCFDA probe as described ^20^. Following incubation with 10 μM H_2_DCFDA for 30 min, cells were washed twice with PBS and scraped into water. The fluorescence of H_2_DCFDA-stained cells was measured with a spectrofluorimeter (excitation wavelength 493 nm, emission wavelength 527 nm). Fatty acids were dissolved in ethanol and added to the medium for 24h.

**Direct evaluation of oxidative damage in the spinal cord by western blot: Carbonyl derivatization with 2,4 dinitrophenylhydrazine.**

Tissues were removed from euthanized mice and flash-frozen on liquid nitrogen. Frozen tissues were homogenized with a Dounce homogenizer, at 4ºC, in the following buffer: 180mM KCl, 5mM MOPS and 3mM EDTA pH 7.3, containing 1μM BHT and a protease inhibitor cocktail (ref 11836153001, Roche Diagnostics GmbH). The homogenates were sonicated for 2 minutes (4ºC, intervals of 10 seconds with continuous pulses at 50% power) and centrifugated for 10 minutes at 1000g and 4ºC. Supernatant was removed and a second step of sonication/centrifugation was performed under the same conditions. Protein concentrations were measured with bicinchroninic acid Protein Assay Kit (Pierce) and 10 micrograms were equalized and loaded on to 10% SDS-PAGE gels. Resolved proteins were transferred to nitrocellulose membranes and derivatized with DNPH as previously described ^37^. After derivatization, membranes were blocked with 5% free fatty acid milk and incubated with monoclonal anti-DNP antibody (dilution: 1/1000, ref D8406, Sigma-Aldrich) for 36 hours at RT. Goat anti-mouse IgG linked to horseradish peroxidase (dilution: 1/10000, ref 2015-08, Dako Denmark) was used as a secondary antibody. Proteins were detected with enhanced chemiluminescence (ECL) western blotting analysis system (ref RPN2135 GE Healthcare). Photographs and quantifications were generated with the Bio-Rad Molecular ImagerTM VersaDocTM MP 4000 System.

**Immunohistochemistry**

Spinal cords were harvested from 22-month-old Wt, *Abcd1^-^* and *Abcd1^-^* fed with the cocktail of antioxidant for 6 months, after perfusion with PFA4%, basically as described ^16, 38^. Spinal cords were embedded in paraffin and serial sections, 5μM thick, were cut in a transversal or longitudinal plane. The sections were stained with haematoxylin and eosin and Sudan black, or processed for immunohistochemistry to glial fibrillary acidic protein (GFAP, Dako, rabbit polyclonal, 1:500), APP (Boehringer, 1:10), synaptophysin (Dako, monoclonal, 1:500), with lectin *Lycopericon esculentum* (Sigma, L-0651, 1:200) used as a marker of microglial cells and 8-oxodG (Abcam, 1:1000). The number of abnormal specific profiles was quantified in every tenth section for each particular stain. Five sections corresponding to the dorsal columns of the cervical spinal cord were analyzed per animal and per stain. Results were expressed as mean values ± standard deviations.

**Behavioural testing**

Treadmill test: The mice were evaluated in five trials in a single-day session. In the first trial, the belt speed was set at 20 cm/s and the inclination at 5º. In the second and third trial, the belt speed was 10 cm/s and the slope was increased to 10º and 20º, respectively. Then, for the fourth and the fifth trials, the inclination was maintained at 20º and the belt speed was increased to 20 and 30 cm/s, respectively. For the three first trials, mice ran 1 minute. For the fourth and fifth tests, time of the experiment was 3 and 7 minutes, respectively. The time between each test was 1, 1, 5 and 20 minutes, respectively. The mice were placed on the top of the already moving belt facing away from the electrified grid and in the direction opposite to the movement of the belt. Thus, to avoid the footshocks, the mice had to locomote forward. Whenever an animal fell off the belt, footshocks were applied for a maximal duration of 1 s.

**Horizontal bar cross test**

The bar cross test was carried out using a wooden bar of 100 cm in length and 2 cm in width (diameter). This bar is just wide enough for mice to stand on with their hind feet hanging over the edge such that any slight lateral misstep will result in a slip. The bar was elevated 50 cm from the bench surface, so that animals did not jump off, yet were not injured upon falling from the bar. The mice are put on one end of the bar and expected to cross to the other end. To eliminate the novelty of the task as a source of slips, all animals were given four trials on the bar the day before and at the beginning of the testing session. In an experimental session, the number of hind limb lateral slips and falls from the bar was counted on four consecutive trials. If an animal fell, it was placed back on the bar at the point at which it fell and was allowed to complete the task. The bar was cleaned with ethanol after each animal ^38^.
