# Supplementary material for: Antioxidants Halt Axonal Degeneration in a Mouse Model of X-Adrenoleukodystrophy
Source: Ann Neurol. 2011 Jul;70(1):84–92. doi: 10.1002/ana.22363 (PMC3229843; doi:10.1002/ana.22363)
Supplement: Supplementary file 5 [file ana0070-0084-SD5.doc]

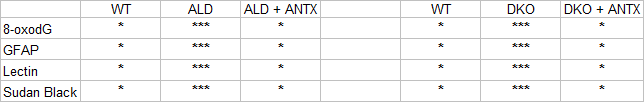


**Supplementary Table.** Summary of the main pathological findings in 1 cm long longitudinal section of the dorsal spinal cord in Wt , *Abcd1*^−^, *Abcd1*^−^ + Antx mice at 22m of age and Wt, *Abcd1*^−^*/Abcd2*^-/-^ , *Abcd1*^−^*/Abcd2*^-/-^ + Antx mice at 18m of age (n=5-6 mice per genotype and condition). DNA damage is stained with 8-oxodG, microglial cells with lectin Lycopericon esculentum and astrocytes with GFAP. *, Normal appearance; **, slight and ***, marked increase in DNA damage or in the number and size of astrocytes and microglia. Sudan black visualizes abnormal lipidic droplets resulting from myelin debris.
